# Petrified Ears: A Clue for Adrenal Insufficiency

**DOI:** 10.3390/dermatopathology8010009

**Published:** 2021-03-06

**Authors:** Sebastiano Recalcati, Fabrizio Fantini

**Affiliations:** Department of Dermatology, ASST Lecco, Alessandro Manzoni Hospital, 23900 Lecco, Italy; f.fantini@asst-lecco.it

**Keywords:** petrified ears, auricular calcification, ectopic calcification, ear reconstruction, metastatic calcification

## Abstract

Petrified ears is an uncommon clinical entity. It describes auricular cartilage hardening, due usually to ectopic calcification or, less commonly, ossification. The most common causes are frostbite and mechanical trauma. However, endocrinopathies have also been reported to cause ectopic calcium deposition through an unknown mechanism. Addison’s disease is the systemic disease most frequently associated, but the exact pathogenesis remains unclear. Patients are usually asymptomatic, and the diagnosis is made incidentally when rigid helices are noted on palpation and can be confirmed by radiological imaging. A biopsy can also help to differentiate between calcification and ossification. The presence of this condition may be a useful clinical sign, which in some cases precedes the development of endocrinopathies by many years. We report on a case, and we review the current literature.

## 1. Introduction

Petrified ears is an uncommon clinical entity: it describes auricular hardening, usually due to ectopic calcification or, less commonly, ossification of the cartilage. The diagnosis is made incidentally when rigid helices are noted on palpation and can be confirmed by radiological imaging. We report on a case showing a challenging surgical reconstruction after oncologic surgery, and we review the current literature.

## 2. Case Report

**Figure 1:**
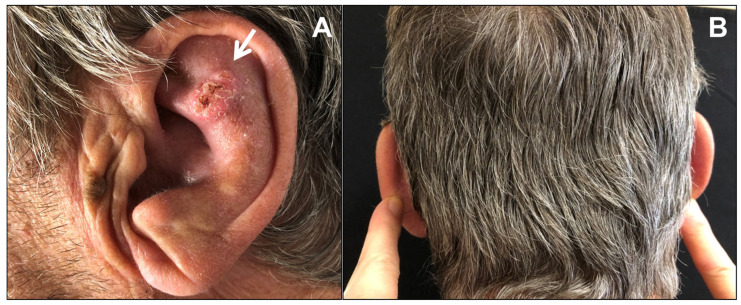
(**A**) Petrified ear with squamous cell carcinoma (arrow); (**B**) Digital probing detects inflexible stiffness in both ears.

**Figure 2:**
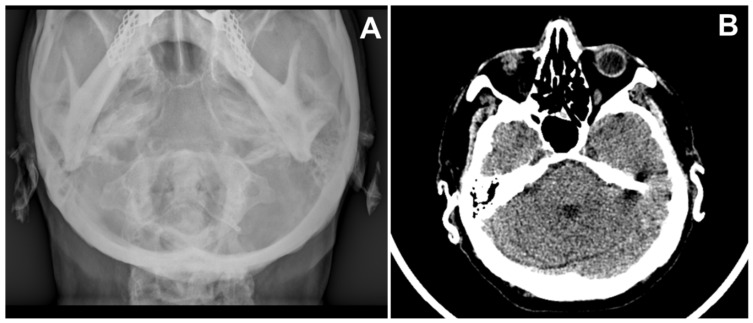
(**A**) X-ray of the skull showing opacification of both helices; (**B**) Computerized tomography of skull reveals calcification of right and left pinna.

**Figure 3:**
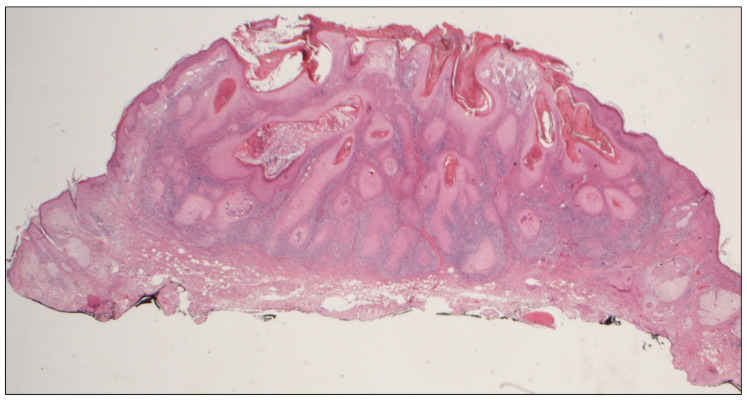
Histologic findings. Invasive, well-differentiated squamous cell carcinoma: keratinizing tumor infiltrating into the dermis (Hematoxylin and eosin, 2.5×).

**Figure 4:**
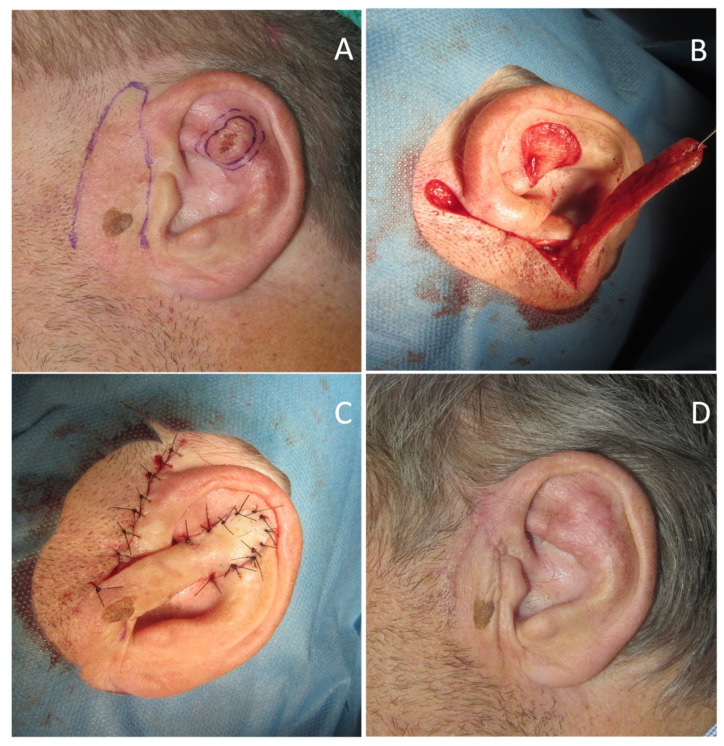
(**A**) Pre-auricular flap design; (**B**) The flap is undermined and transposed; (**C**) At the end of the first stage the distal end of the flap is sutured to the recipient site; (**D**) Final result at two months.

A 71-year-old man was referred for surgery for a squamous cell carcinoma of the left scapha (Figure 1A). He recalled progressive stiffening of both ears for 20 years, associated with pain exacerbated in bed at night, and complained of hearing loss. He denied a history of ear trauma, frostbite, or inflammatory disorders. He had an 18-year history of primary hypothyroidism and primary adrenal insufficiency, currently treated with hydrocortisone and levothyroxine. At palpation, both ears were stony hard, inflexible, and unmalleable (Figure 1B), without any visible external change, apart from the carcinoma. No alterations of the nasal ala were observed. A radiograph (Figure 2A) and a computerized tomography scan (CT) showed calcification of the entire auricular cartilage with sparing of the earlobes (Figure 2B). Full blood cell count, renal biochemistry, calcium, phosphorus, glucose, uric acid, thyroid function, and parathyroid hormone tests revealed normal findings. A diagnosis of petrified ears was made. No specific treatment of the calcification was performed due to the mild symptom entity. The squamous cell carcinoma was surgically excised to the calcified plane, with negative surgical margins (Figure 3). Reconstruction of the surgical defect was accomplished with a two-stage interpolated, inferiorly based, pre-auricular transposition flap (Figure 4). Immediately after the excision, the flap was incised and undermined, and its distal end was transposed and sutured to the ear defect. After 3 weeks, the pedicle of the flap was severed, and remodeling and final suturing were performed. The donor site was primarily closed.

## 3. Discussion

Petrified ear is a rare diagnosis, which was first reported in 1866 by the Czech anatomist Vincent Bochdalek [1]. To date, there have been approximately 150 cases of petrified ear reported worldwide. It is characterized by the development of stony-hard auricular cartilage of one or both the ears. Petrification is most often due to calcification, with amorphous, insoluble calcium salts deposited into tissue. Rarely, petrification can be due to ossification, whereby soft tissue is converted into bone, which would not normally otherwise occur [2]. The calcification may be metastatic in patients with elevated serum levels of calcium and phosphorus or dystrophic when occurring after damage or inflammation of the cartilage. Common causes are frostbite and mechanical trauma; however, endocrinopathies have also been reported to cause ectopic calcium deposition. In this group, Addison’s disease is the most frequently associated disease, but diabetes mellitus, hypothyroidism, and acromegaly were also reported [3,4,5,6]. The presence of ear calcification may be a useful clinical sign, which may precede the development of endocrinopathies by many years. The exact pathogenesis remains unclear. Diagnosis is made incidentally when rigid helices are noted on palpation. Blood tests should be requested to investigate possible endocrinopathies, such as Addison’s disease or hypopituitarism and conditions that pre-dispose to hypercalcemia. X-ray and CT scans will show dense opacities within the cartilage [7]. Histology will show the deposition of calcium in the cartilage, or rarely ossification, with fibrocartilaginous tissue being replaced by lamellar bone. Most patients with petrified ears are asymptomatic. Some may report external otalgia and discomfort when sleeping on the affected side. Patients can develop symptomatic conductive hearing loss due to impediment of cerumen and ossification of the external ear canal [8]. A unique presentation of petrified ears with pain due to fracture was reported [9]. In asymptomatic patients, no specific therapy is required. Wedge resection of the affected cartilage or conchal reduction surgery has yielded improvement in some symptomatic patients [10].

Reconstruction of the auricle after oncologic surgery is often complex due to the thin, adherent skin overlying a cartilaginous frame. In petrified ears, reconstructive surgery is particularly challenging because the calcified, stiff cartilaginous frame makes some reconstructive options (such as second intention healing, skin graft, transcartilaginous flaps) impractical. Even if two surgical steps were required, we considered that an interpolated transposition flap would be minimally at risk as to the vascular supply. The pre-auricular skin was chosen as the donor site because the mastoid region would have required either a trans-auricular tunnelization or, alternatively, an excessive bridging of the flap above the auricle. The final cosmetic result was excellent.

## 4. Conclusions

Underlying systemic diseases, especially adrenal insufficiency, should be considered when patients present with petrified ear.
